# The PPE2 protein of *Mycobacterium tuberculosis* translocates to host nucleus and inhibits nitric oxide production

**DOI:** 10.1038/srep39706

**Published:** 2017-01-10

**Authors:** Khalid Hussain Bhat, Shruti Srivastava, Sandeep Kumar Kotturu, Sudip Ghosh, Sangita Mukhopadhyay

**Affiliations:** 1 Laboratory of Molecular Cell Biology, Centre for DNA Fingerprinting and Diagnostics (CDFD), Tuljaguda Complex, Nampally, Hyderabad, India; 2 Graduate Studies, Manipal University, Manipal, Karnataka, India; 3 Molecular Biology Division, National Institute of Nutrition (ICMR), Jamai-Osmania PO, Hyderabad, India

## Abstract

*Mycobacterium tuberculosis*, the bacterium that causes tuberculosis, is one of the most successful pathogens of humans. It has evolved several adaptive skills and evasion mechanisms to hijack the immunologically educated host to suit its intracellular lifestyle. Here, we show that one of the unique PPE family member proteins of *M. tuberculosis*, PPE2, can limit nitric oxide (NO) production by inhibiting *inos* gene transcription. PPE2 protein has a leucine zipper DNA-binding motif and a functional nuclear localization signal. PPE2 was translocated into the macrophage nucleus *via* the classical importin α/β pathway where it interacted with a GATA-binding site overlapping with the TATA box of *inos* promoter and inhibited NO production. PPE2 prolonged intracellular survival of a surrogate bacterium *M. smegmatis in vitro* as well as *in vivo*. This information are likely to improve our knowledge of host-pathogen interactions during *M. tuberculosis* infection which is crucial for designing effective anti-TB therapeutics.

*Mycobacterium tuberculosis* is a successful intracellular pathogen that has developed several adaptive skills and evasion mechanisms to hijack host defense processes^1^. Complete genomic sequencing of *M. tuberculosis* H37Rv genome revealed the presence of two novel gene families that comprise almost 10% of the coding capacity of the genome^2^,^3^. These are designated as *pe* and *ppe* genes and are characterized by the presence of conserved Proline-Glutamate (PE) and Proline-Proline-Glutamate (PPE) residues near the start of their coding sequences respectively^2^,^4^. These genes are found to be unique to Mycobacterium genus, both pathogenic and non-pathogenic but are particularly abundant in pathogenic species such as *M. tuberculosis*^2^. Though initially speculated to be variable antigens, these genes appear to be critical modulators of host responses by employing diverse mechanisms and appear to be associated with the evolution of mycobacterial pathogenicity^2^,^3^.

A NCBI conserved domain database search predicted PPE2 to be a member of the orthologous group COG5651, members of which are involved in cell motility and secretion^5^,^6^. While analyzing the sequences of the PPE family proteins of *M. tuberculosis*, we identified presence of a monopartite nuclear localization signal (NLS) at the C-terminal region (473–481 aa) and a leucine zipper motif (319–340 aa) in PPE2 protein, typical to eukaryotic transcription factors. Several plant pathogenic bacterial effector proteins are known to be targeted to different subcellular organs including the nucleus and most of the nucleus targeted proteins are characterized by the presence of functional NLSs^7^. For example, VirD2 and VirE2 proteins of *Agrobacterium tumefaciens* contain bipartite NLS which are secreted into host cells via the type IV secretion system. VirD2 binds the *A. thaliana* importin-α subunit KAPα1 in a manner dependent on the presence of the C-terminus-proximal NLS that utilizes the classical nuclear import pathway^8^. Similarly, AvrBs3, AvrXa5, AvrXa7, AvrXa10 and PthA proteins of *Xanthomonas* spp., contain monopartite NLS which allows them to be localized into the plant nucleus and regulate plant gene expression during infection^7^,^9^. These transcription activator-like (TAL) effectors are characterized by a central DNA-binding region consisting of a nearly identical tandem of 34 amino acid repeats, followed by a classic monopartite NLS and an acidic transcriptional activation domain^9^. Nuclear targeting of bacterial effector proteins and subsequent pathology of the host cells appears to be an emerging pathogenic mechanism of bacteria^7^,^9^,^10^. However, in animals, few bacterial proteins are known to be targeted to the nuclei though bioinformatics analyses predicted many pathogenic bacterial proteins to contain putative NLSs, but in most cases, their pathophysiological functions are yet to be demonstrated^10^,^11^. Accordingly, we speculated nuclear localization of PPE2 where it may modulate transcription by binding to the regulatory regions of host genes.

Expression of PPE2 is up-regulated in *M. tuberculosis* during hypoxia and nitric oxide (NO) stress as well as in *DosS*-null mutants upon exposure to NO^12^,^13^ and also when resident inside the macrophage environment^13^. Interestingly, homologs of PPE2 are absent in non-pathogenic mycobacteria but a homolog of PPE2 present in *M. leprae* (PPE12) lacks the predicted NLS or DNA-binding domain as found in *M. tuberculosis* PPE2. This indicates a possible role of this protein in protecting the bacterium during NO stress. Indeed, earlier we found that PPE2 is a secretory protein and in infected macrophages, PPE2-null mutants allowed higher production of nitric oxide^14^. The potential mechanisms by which NO may affect antimicrobial activity are protean. NO and other RNI can modify bacterial DNA, protein, and lipids at both the microbial surface and intracellularly. NO can deaminate as well as directly damage bacterial DNA by generating abasic sites and strand breaks^15^. Though, the role of NO in human infection is debatable, several lines of evidences suggest that NO plays a contributory role in human host defense against tuberculosis^1^,^16^. At low pH, NO bactericidal effects are shown to be boosted^16^. Peripheral blood monocytes and alveolar macrophages from the lungs of patients with active pulmonary tuberculosis express higher levels of iNOS^17^,^18^. Interestingly, it has been observed that *in vitro* tolerance of mycobacteria to NO/ROIs is strain-, dose- and time-dependent, with pathogenic strains being more resistant than non-pathogenic stains^16^. Therefore, it appears that pathogenic strains may employ certain defensive strategies to counter the toxic effects of NO and its different oxidation products. Our earlier studies demonstrated that macrophages infected with PPE2-null mutant *M. tuberculosis* can produce higher levels of NO as compared to macrophages infected with wild-type strain^14^ suggesting that PPE2 may help the bacteria to inhibit NO production and could be a virulent factor. In this manuscript, we found that PPE2 mimics a eukaryotic transcription factor that translocates into the nucleus and binds to upstream regulatory sequences of iNOS, modulating transcription driven by its promoter.

## Results

### PPE2 contains a functional NLS sequence important to translocate to nucleus

**Figure 1:**
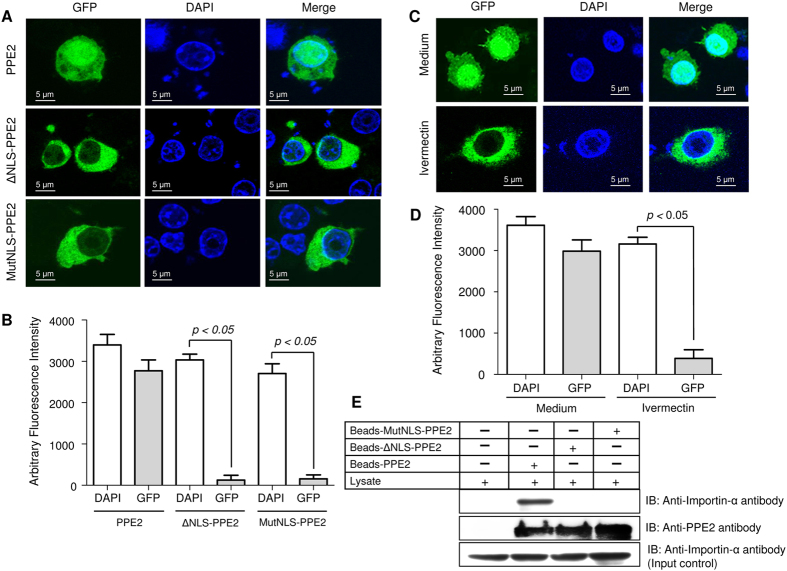
PPE2 contains a functional nuclear localization signal. (**A**) EGFP-tagged PPE2 protein (green) with mutated NLS failed to translocate into the nucleus. RAW 264.7 macrophages were transfected with either EGFP-tagged wild-type PPE2 (EGFP-PPE2) or PPE2 with truncated NLS (EGFP-ΔNLS-PPE2) or PPE2 with mutated NLS (EGFP-MutNLS-PPE2) and examined by confocal microscopy after at 24 hours post-transfection. Nuclei were stained with DAPI (Blue). (**B**) Image quantification of nuclear import of PPE2 was carried out in cells (n = 24) transfected with EGFP-tagged either wild-type PPE2 (EGFP-PPE2) or PPE2 with truncated NLS (EGFP-ΔNLS-PPE2) or PPE2 with mutated NLS (EGFP-MutNLS-PPE2) using ImageJ software. (**C**) EGFP-tagged PPE2 failed to be translocated to the nucleus in the presence of ivermectin, a classical nuclear import inhibitor. RAW 264.7 cells were transfected with EGFP-PPE2 (green) followed by treatment with 10 μM ivermectin or vehicle controls and examined under a confocal microscope at 24 hours post-transfection. Nuclei were stained with DAPI (Blue). (**D**) Image quantification of nuclear import of PPE2 in RAW 264.7 macrophages was carried out using ImageJ software. Twenty cells per experimental sample were taken for quantification of nuclear import of PPE2 in RAW 264.7 macrophages. (**E**) The NLS of PPE2 interacts with importin-α. Talon-bound 6X-histidine-tagged wild-type PPE2, MutNLS-PPE2 and ΔNLS-PPE2 were incubated with RAW 264.7 lysates overnight and after washing, the bound proteins were probed by Western blotting using anti-importin-α antibody or anti-PPE2 antibody. About 50 μg of the cell extract from each group was used as input control. All data are representative of 3 independent experiments.

The sequence analysis of PPE2 (Rv0256c) predicted a strong monopartite nuclear localization signal as well as presence of a leucine zipper motif at the C-terminal region of PPE2 with 100% probability of nuclear transport (Fig. S1). We found that the monopartite NLS present in PPE2 is biologically functional, since transiently expressed GFP-tagged PPE2 in RAW 264.7 macrophages could be localized into the nucleus, whereas truncated mutants without the NLS signal (ΔNLS-PPE2) failed to do so (Fig. 1A,B). Similarly, when the positively charged arginine residues in the monopartite NLS were replaced by neutral alanine residues, the mutant PPE2 (MutNLS-PPE2) also failed to be localized inside the nucleus.

Nuclear import of PPE2 involved classical importin α/β since ivermectin^19^ was able to block its nuclear import (Fig. 1C,D) and PPE2 with intact NLS sequence directly interacted with importin α/β but not ΔNLS-PPE2 or MutNLS-PPE2 (Fig. 1E). These data suggest that PPE2 translocates to the nucleus by utilizing the traditional nuclear import pathway like VirD2 of *Agrobacterium*^7^,^8^,^9^, AvrBs3 of Xanthomonas^7^,^9^ rather than a ‘piggyback’ mechanism often utilized by some proteins lacking typical NLS like IκBα^20^ for their nuclear import.

### Nuclear translocation of PPE2 is important for inhibition of nitric oxide in activated macrophages

**Figure 2:**
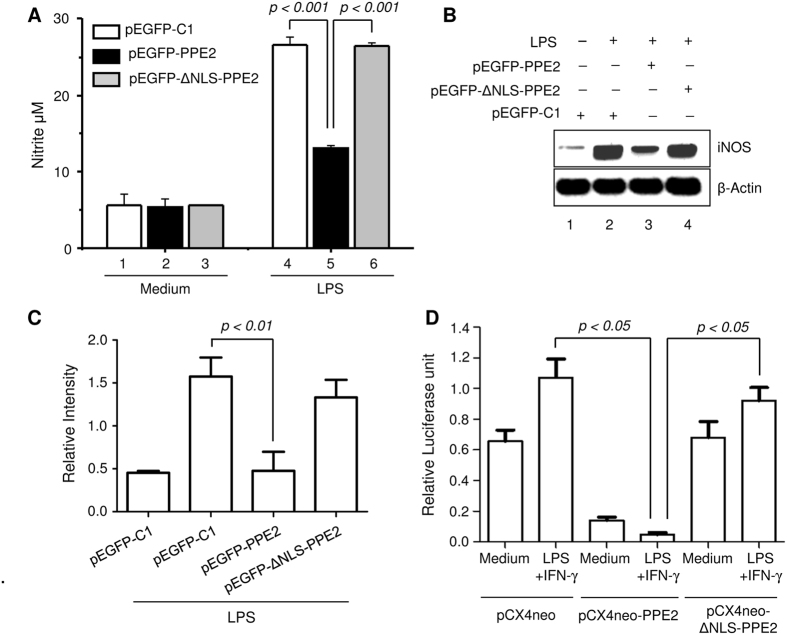
PPE2 inhibits nitric oxide (NO) production in macrophages. (**A**) PPE2 with intact NLS inhibits nitrite accumulation in macrophages. RAW 264.7 macrophages were transfected with either pEGFP-C1 vector (control) or pEGFP-PPE2 or pEGFP-ΔNLS-PPE2. After 8 hours of transfection, the cells were either left untreated and cultured in medium alone or stimulated with 5 μg/ml LPS. After 48 hours, nitrite accumulation was measured in the culture supernatants using Griess reagent. (**B**) RAW 264.7 macrophages were transfected as described above and after 2 hours post-stimulation with LPS, total RNA was extracted to perform semi-quantitative RT-PCR using *inos*-specific primers. β-actin was used as control. (**C**) Densitometric analysis of *inos* mRNA expression in macrophages. The graph summarizes densitometric analysis of 3 independent experiments. The results shown are expressed as the ratio of iNOS to β-actin. (**D**) PPE2 inhibits luciferase expression driven by *inos* promoter. Stably transfected RAW 264.7 macrophages harbouring an empty vector (pCX4neo) or wild-type PPE2 (pCX4neo-PPE2) or PPE2 with truncated NLS (pCX4neo-ΔNLS-PPE2) were co-transfected with luciferase gene under the control of *inos* promoter (*inos-luc*) and β-Galactosidase driven by a constitutive CMV promoter. Cells were either cultured in medium alone or stimulated with LPS + IFN-γ for 24 hours. The relative expression of *inos-*driven luciferase was normalized to β-Galactosidase expression. Data were expressed as mean ± SD of 3 independent experiments.

Earlier, we found that in infected macrophages, PPE2-null mutants allowed higher production of nitric oxide^14^, indicating a role of PPE2 in inhibiting NO production. Macrophages expressing wild-type PPE2 could significantly inhibit formation of LPS-stimulated nitrite, but not the cells transfected with ΔNLS-PPE2 or MutNLS-PPE2 (Fig. 2A). Since NO is predominantly produced by the inducible nitric oxide synthase (iNOS) in macrophages, semi-quantitative RT-PCR was performed to compare LPS-induced *inos* transcript levels in these groups and PPE2 was found to strongly inhibit *inos* gene transcription (Fig. 2B,C). When a luciferase reporter gene driven by the *inos* promoter was transfected to RAW 264.7 macrophages stably expressing wild-type PPE2 (pCX4Neo-PPE2) (Fig. S2), luciferase activity was found to be significantly inhibited upon stimulation with LPS as compared to those cells harbouring truncated PPE2 (pCX4Neo-ΔNLS-PPE2) (Fig. 2D), suggesting a role of PPE2 in inhibiting transcription from the *inos* promoter.

### PPE2 binds to iNOS promoter

**Figure 3:**
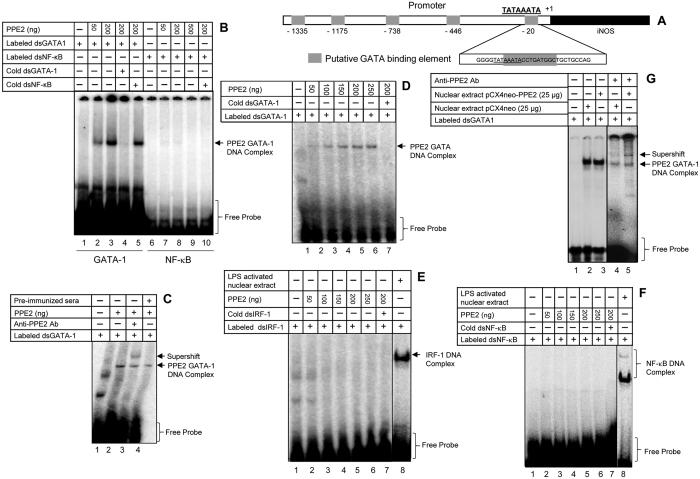
PPE2 specifically interacts with GATA-1-binding element proximal to the *inos* TATA box. (**A**) A schematic diagram of putative GATA-1-binding consensus elements present in the *inos* promoter. The proximal GATA-1-binding element is overlapped with the TATA box. (**B**) PPE2 interacts with the proximal GATA-1-binding element. Different concentrations of recombinant PPE2 protein were incubated with labelled double stranded (ds) oligonucleotides representing the cognate proximal GATA-1 binding element or NF-κB-binding elements and the DNA-protein complexes were resolved by EMSA. In cold competition reactions, 100-fold molar excess unlabelled double stranded-oligonucleotides were used. (**C**) A supershift was observed, when the EMSA reactions (150 μg of PPE2 protein) were pre-incubated with anti-PPE2 antibody (10 μg/reaction). (**D**) PPE2 also specifically binds to consensus GATA-binding element. Different concentrations of recombinant PPE2 protein were incubated with labelled ds-oligonucleotides representing a consensus GATA-1-binding element and the DNA-protein complexes were resolved by EMSA. In cold competition reactions, 100-fold molar excess unlabelled ds-oligonucleotides were used. (**E** and **F)**. Recombinant PPE2 did not interact with consensus dsIRF-1 binding element (**E**) or consensus dsNF-κB-binding element (**F**) as determined by EMSA. **(G)** PPE2 present in the nuclear extract prepared from a stably transfected RAW 264.7 macrophages harbouring pCX4Neo-PPE2, binds specifically to the proximal GATA-1 element present in the *inos* promoter. A supershift was observed in nuclear extract prepared from stable cells expressing PPE2 when incubated along with anti-PPE2 antibody (lane 5). All the results shown are representative of 3 independent experiments.

As PPE2 was predicted to contain a leucine zipper DNA-binding motif, we speculated that PPE2 probably binds to some crucial regulatory element of the promoter important for *inos* gene transcription. In addition to major role played by NF-κB and IRF-1^21^, binding sites of several other transcription factors linked to iNOS regulation are found to be conserved that include GATA-1^22^. Using Alibaba2.1 (http://wwwiti.cs.uni-magdeburg.de/-grabe/alibaba2)^23^, we found at least five putative GATA-1 binding sites in the 5′-upstream region of the transcriptional start site. Interestingly, one of the putative sequences (−16 to −25) was found to be overlapping with the TATA box close to the transcription initiation site (Fig. 3A). We observed a specific and concentration-dependent binding of recombinant PPE2 protein to the GATA-1-binding oligonucleotide proximal to the TATA box of *inos* promoter (Fig. 3B). Specificity of binding was further confirmed by a supershift using anti-PPE2 antibody (Fig. 3C, lane 3). PPE2 could also specifically interact with a consensus GATA element (Fig. 3D) but failed to interact with cognate oligonucleotides to IRF-1 (Fig. 3E) as well as NF-κB (Fig. 3F). We also observed specific binding of PPE2 with the GATA-1 binding oligoneucleotide proximal to the *inos* TATA box when nuclear extract prepared from the stable cells expressing wild-type PPE2 (mostly translocated to the nucleus (Fig. S2)) was used in EMSA (Fig. 3G). A supershift was observed when anti-PPE2 antibody was incubated with these nuclear extracts (Fig. 3G, lane 5) but not with those of macrophages harbouring the backbone vector indicating that PPE2 when present inside nucleus can interact with cognate oligonucleotides to GATA-1 (Fig. 3G, lane 3). Retarded bands were also observed in control extracts (Fig. 3G, lanes 2 and 4) presumably due to presence of native GATA-1 protein in the control extracts.

### PPE2 was responsible for better survival of the bacilli inside the cells

**Figure 4:**
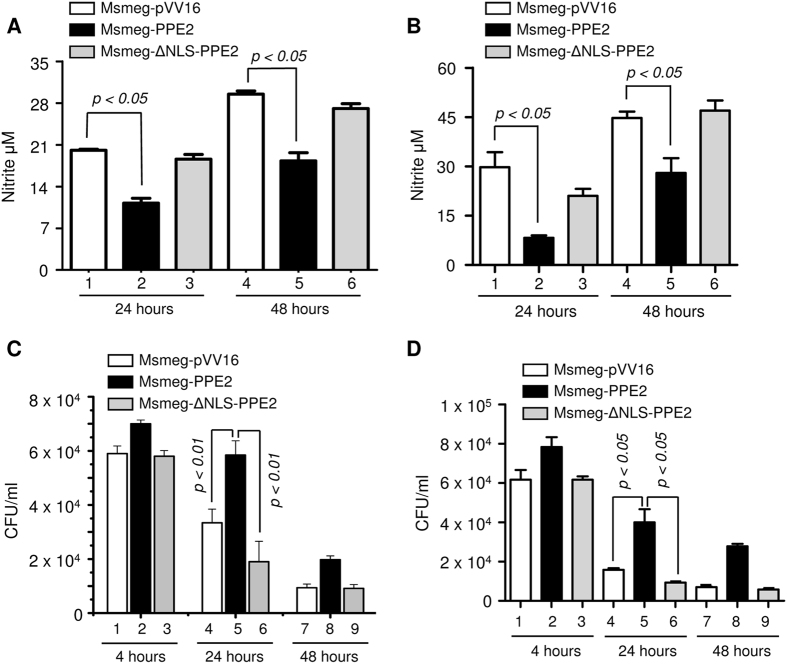
*M. smegmatis* expressing PPE2 with intact NLS inhibited NO production and survived significantly longer inside macrophages. About 0.5 million peritoneal macrophages from C57BL/6 mice (**A** and **C**) or RAW 264.7 macrophages (**B** and **D**) were infected with either *M. smegmatis* harbouring pVV16 vector alone (Msmeg-pVV16), or pVV16-PPE2 (Msmeg-PPE2) or pVV16-ΔNLS-PPE2 (Msmeg-ΔNLS-PPE2) at 1:10 multiplicities of infection (MOI) and supernatants were removed for quantification of nitrite accumulation at 24 and 48 hours (**A** and **B**). The infected cells were lysed at 4, 24 and 48 hour post-infection. The cell lysates were plated on 7H10 plates supplemented with 10% ADC and 0.05% of Tween 80 and incubated for 4 days for counting the number of colony forming units (CFUs) (**C** and **D**). Data were expressed as mean ± SD of 3 independent experiments.

**Figure 5:**
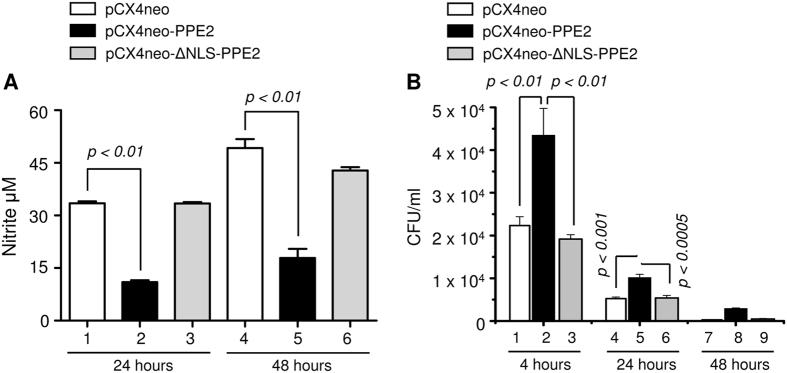
*M. smegmatis* survived significantly longer inside RAW 264.7 macrophages stably expressing PPE2 with intact NLS. Stably transfected RAW 264.7 macrophages (0.5 × 10^6^) harbouring either the vector alone (pCX4Neo) or pCX4Neo-PPE2 or pCX4Neo-ΔNLS-PPE2 were infected with *M. smegmatis* 1:10 MOI and nitric oxide estimation was carried out at 24 and 48 hours (**A**) and CFUs were counted at 4, 24 and 48 hours post-infection (**B**). Data were expressed as mean ± SD of 3 independent experiments.

Next, we examined whether migration of PPE2 in the nucleus and reduction in NO production in turn facilitated intracellular survival of the bacilli inside macrophages. When peritoneal macrophages from C57BL/6 mice and RAW 264.7 murine macrophages were infected with Msmeg-PPE2, they produced significantly low levels of NO as compared to cells infected with *M. smegmatis* expressing either ΔNLS-PPE2 (Msmeg-ΔNLS-PPE2) or backbone vector pVV16 (Fig. 4A,B). Also there is an inverse correlation between the levels of NO production and bacterial survival inside macrophages as Msmeg-PPE2 survived for significantly longer time period inside both the peritoneal macrophages (Fig. 4C) and RAW 264.7 macrophages (Fig. 4D) producing lower levels of NO as compared to Msmeg-ΔNLS-PPE2 or Msmeg-pVV16. Similarly, *M. smegmatis* survived significantly longer in RAW 264.7 macrophages stably expressing wild-type PPE2 but not ΔNLS-PPE2 (Fig. 5B) and this observation was well correlated with the levels of NO produced in these cells. Upon infection, the RAW 264.7 macrophages stably expressing wild-type PPE2 produced significantly lower amount of NO as compared to macrophages expressing ΔNLS-PPE2 (Fig. 5A).

Lower expression may be attributed to the presence of translocated PPE2 in the nucleus which is presumably leaked into cytoplasm from the arrested endosomes^24^,^25^ containing the invading mycobacteria. To test this hypothesis, we examined localization of PPE2 in infected macrophages over a period of time. In RAW 264.7 macrophages infected with *M. smegmatis* expressing wild-type PPE2 (Msmeg-PPE2), PPE2 was found to be colocalized with an early endosome marker EEA1 at around 1 hour post-infection, but at 6 hour post-infection, most of the PPE2 protein was found to be localized in cytoplasm as evident from its co-localization with a cytoplasmic marker tubulin (Fig. S3A). The leaked cytoplasmic PPE2 then translocates to the nucleus as we also observed prominent PPE2 staining along with DAPI (a nuclear marker) at 6 hour post-infection (Fig. S3). As expected, no PPE2-specific staining was detected in cells infected with *M. smegmatis* harbouring the backbone vector pVV16 (Msmeg-pVV16) (Fig. S3B). We further confirmed presence of PPE2 in the nucleus of C57BL/6 peritoneal macrophages infected with Msmeg-PPE2 by Western blotting (Fig. S4A) and confocal microscopy (Fig. S4B).

**Figure 6:**
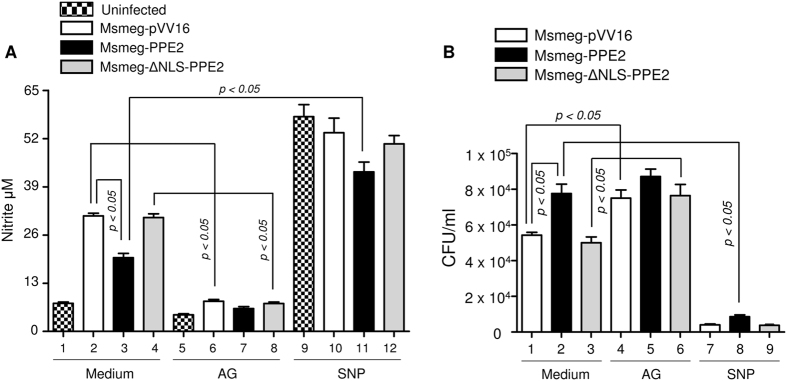
Effect of NO inhibitor, aminoguanidine (AG) and NO donor sodium nitroprusside (SNP) on survival of *M. smegmatis* harbouring either backbone vector (Msmeg-pVV16), or expressing PPE2 (Msmeg-PPE2) or ΔNLS-PPE2 (Msmeg-ΔNLS-PPE2). Murine peritoneal macrophages (0.5 × 10^6^) were either cultured in medium alone or pre-treated with either AG (300 μg/ml) or SNP (100 μM) followed by infection with either Msmeg-pVV16, or Msmeg-PPE2 or Msmeg-ΔNLS-PPE2 at 1:10 multiplicities of infection (MOI). Twenty four hours after infection, supernatants were removed for nitrite determination and the cells were lysed for CFU assay. The cell lysates were plated on 7H10 plates supplemented with 10% ADC, Kanamycin (25 μg/ml), Hygromycin B (50 μg/ml) and 0.05% of Tween 80 and incubated for 4 days for counting the number of colony forming units (CFUs). Data were expressed as mean ± SD of 3 independent experiments.

NO produced by the activated macrophages plays an important role as a cytotoxic effector molecule against various pathogens including mycobacteria at least in mice model of infection^1^,^15^, though its role in human infection is highly debatable^16^. To understand a direct role of NO in the survival of *M. smegmatis*, we next pre-treated the C57BL6 peritoneal macrophages with either sodium nitropruside (SNP), a known NO donor (or aminoguanidine (AG), an inhibitor of nitric oxide synthase^26^,^27^ before infection with Msmeg-PPE2 or Msmeg-ΔNLS-PPE2 or Msmeg-pVV16 and intracellular survival of these strains were examined. The control cells were left in medium alone. As expected, Msmeg-PPE2 survived better than Msmeg-ΔNLS-PPE2 or Msmeg-pVV16 in untreated cells which also correlated well with the levels of NO induced during infection (Fig. 6). However, in the presence of high levels of NO in SNP-treated cells, all the recombinant strains showed poorer CFU counts whereas inhibition of NO production in macrophages by AG allowed survival of Msmeg-ΔNLS-PPE2 and Msmeg-pVV16 similar to Msmeg-PPE2 (Fig. 6). These results indicate that NO was responsible for the killing of mycobacteria in macrophages. Therefore, it appears that mycobacteria evolved strategies to check NO production in macrophages by targeting *inos* using PPE2 protein.

**Figure 7:**
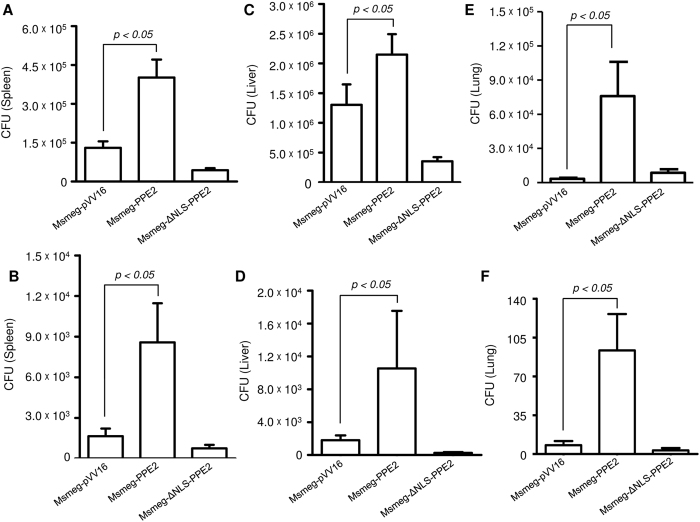
*M. smegmatis* expressing PPE2 with intact NLS had better survival in infected mice. (**A**) C57BL/6 mice (n = 6) were infected intraperitoneally with 50 × 10^6^ *M. smegmatis* harbouring pVV16 vector alone (Msmeg-pVV16), or pVV16-PPE2 (Msmeg-PPE2) or pVV16-ΔNLS-PPE2 (Msmeg-ΔNLS-PPE2) and sacrificed to collect spleen, liver and lung. The number of CFUs was counted from the spleen (**A** and **B**), liver (**C** and **D**) and lung (**E** and **F**) homogenates collected at 2 day post-infection (**A,C** and **E)** and 7 day post-infection (**B,D** and **F**). Data represents mean ± SD of 6 mice per group for each time point.

**Figure 8:**
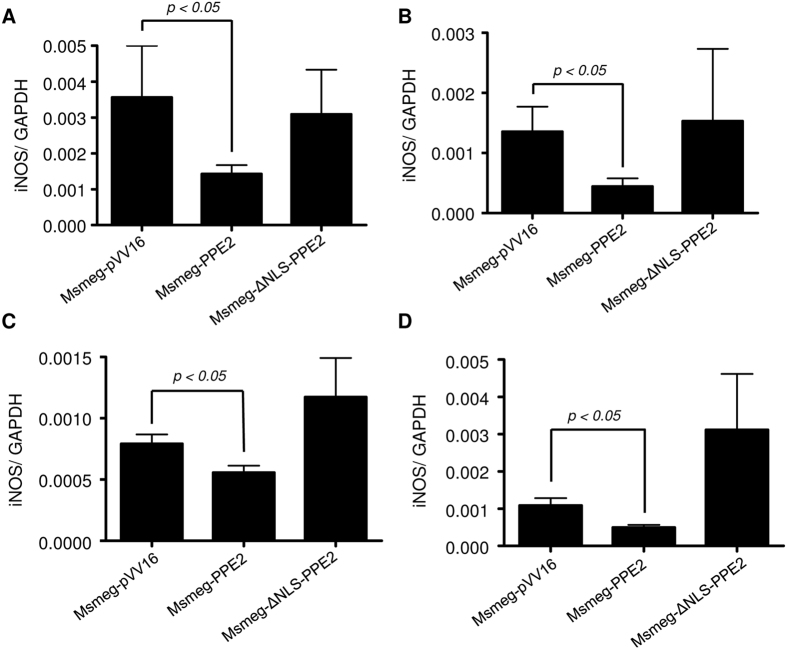
*M. smegmatis* expressing PPE2 inhibits *inos* expression in infected mice. C57BL/6 mice were infected intraperitoneally with 50 × 10^6^ *M. smegmatis* harbouring control vector pVV16 (Msmeg-pVV16) or pVV16-PPE2 (Msmeg-PPE2) or pVV16-ΔNLS-PPE2 (Msmeg-ΔNLS-PPE2) and at day 2 and day 7 post-infections, the mice were sacrificed and total RNA was isolated from lung and spleen. The levels of *inos* mRNA were measured by reverse-transcription qPCR and the expression levels were normalized to corresponding *gapdh* levels in lungs at 2 day post-infection **(A)** and 7 days post-infection **(B)** as well as in spleen at 2 day post-infection **(C)** and 7 day post-infection **(D)**.

Next, C57/BL6 mice were infected intraperitoneally with either Msmeg-PPE2 or Msmeg-ΔNLS-PPE2 or Msmeg-pVV16 and bacterial loads as well as iNOS induction was quantitated. Bacterial loads were found to be significantly higher in spleen, liver and lungs in mice infected with Msmeg-PPE2 as compared to mice infected with Msmeg-ΔNLS-PPE2 or Msmeg-pVV16 at both day 2 (Fig. 7A,C and E) and day 7 (Fig. 7B,D and F). The increased bacterial load in Msmeg-PPE2-infected mice was found to be associated with decreased levels of *inos* expression in these mice whereas opposite was found to be true for Msmeg-ΔNLS-PPE2- or Msmeg-pVV16-infected control mice (Fig. 8). These data suggest an important role of PPE2 in inhibiting iNOS/NO induction and conferring survival advantages to mycobacteria.

## Discussion

The studies presented herein hint a putative pathophysiological role of *M. tuberculosis* protein, Rv0256c (PPE2), which inhibits the production of iNOS at the transcriptional level and thus NO production in macrophages. Inhibition of NO production could be one of the mechanisms by which the bacilli create a favorable environment inside the macrophages to favor their survival. Although tuberculosis is multifactorial and there is no single factor reported to be exclusively responsible for the pathogenicity of *M. tuberculosis*, a wealth of literature argues the importance of NO in the killing of *M. tuberculosis*.

We reported earlier that PPE2 is expressed during active pathogenesis of *M. tuberculosis*^28^. Our studies indicate that PPE2 contains a physiologically active NLS as well as a DNA-binding leucine zipper motif that translocates into host cell nucleus and inhibits gene transcription driven by the *inos* promoter and promotes better survival of *M. smegmatis* expressing PPE2 *in vitro* as well as *in vivo* (Figs 1, 2, 4, 5 and 7; Fig. S5). Upon infection, PPE2 appears to be localized in endosomes earlier during infection, but later on was found to be translocated to the nucleus probably by leaking into the cytosol and then binding to importin-α via its NLS. Several mycobacterial proteins are known to escape into the cytosol from the arrested phagolysosome^29^,^30^. Another possibility is that mycobacteria themselves can escape directly into the cytosol from phagolysosomes to release virulent proteins for modulating host functions^31^,^32^,^33^. Interestingly, PE_PGRS62^34^, PE5 and PE15^35^ can also inhibit *inos* gene transcription in macrophages. However, the mechanisms are not well understood. Though the bacterium lacks classical virulence factors such as toxins, typical to several other bacterial pathogens, it employs vast array of factors to modulate multiple host cellular processes to favor its intracellular survival^1^. The ability to modulate host cellular processes through functional mimicry is a powerful tool, usually achieved through homologous enzymes that have been subverted for the benefit of pathogen and often have considerable similarities with the cognate host proteins at the primary amino acid sequence levels particularly around their active or regulatory sites^36^,^37^. Typical examples are serine threonine kinase YpkA, cysteine protease YopJ from *Yersinia pseudotuberculosis*^38^ and also inositol phosphatase SopB from *Salmonella dublin*^39^. Interestingly, we did not find any significant sequence homology of PPE2, a leucine zipper protein, with zinc-finger containing GATA-binding factor, the cognate target DNA sequence of which it specifically binds, suggesting a functional mimicry at the tertiary level. Such convergent evolution is perhaps intriguing as it requires sculpting the available genes and proteins encoded by the bacteria to perform a new function rather than obtaining a foreign homologous sequence through horizontal gene transfer^36^,^37^. Since a GATA-1 consensus sequence was present overlapping with *inos* TATA box, we speculate that PPE2 protein possibly sterically inhibit recruitment of transcription machinery by directly competing with binding of TATA binding protein. Such direct competition mechanism of transcriptional repression is widely prevalent in bacteria such as the LacI repressor protein of *lac* operon^40^. However, inhibition of transcription by direct competition is though demonstrated in eukaryotes, their ability to do so under physiological conditions is arguable. For instance, chicken ovalbumin upstream promoter transcription factors (COUP-TFI and II, also known as Ear 2 and 3) are widely conserved orphan nuclear hormone receptors that have binding specificity similar to those of RAR, VDR, and SF-1 receptors^41^. When COUP-TF factors are overexpressed in transfection assays, activation by SF-1 of vertebrate CYP17 promoter is found to be inhibited^42^. However, during MHC-I promoter repression, COUP-TFII antagonizes the activity of NF-κB from non-overlapping sites, suggesting a mode of repression that is independent of direct competition^43^. Therefore, alternative mechanisms in which PPE2 may inhibit transcription by binding to non-overlapping GATA-1 sites present in the upstream region of the *inos* promoter cannot be rule out. It will be interesting to further investigate in this direction.

## Methods

### Bacterial strains

The *Mycobacterium smegmatis* strain mc^2^155 was a kind gift from Prof. Dipankar Chatterji, Indian Institute of Science, Bengaluru, India and maintained in 7H9 medium supplemented with 0.05% Tween-80 and 10% ADC (HIMEDIA, India).

### *In silico* analyses of PPE2

Overall structural and functional predictions were carried out using online PredictProtein server (http://www.predicprotein.org)^44^. Prosite scan was performed to identify presence of conserved functional domains in PPE2 protein using online tools available at the URL pointer http://expasy.org/prosite/. NCBI conserved domain database was used to look for the presence of conserved domains^5^. Alibaba2.1 software was used to predict the putative transcription factor binding sites in mouse *inos (nos2*) promoter sequence^23^.

### Cloning, expression and purification of PPE2

The PPE2 open reading frame was amplified from a bacterial artificial chromosome (BAC) clone BAC-Rv329 (A5), a kind gift from Dr. Shekhar C. Mande, NCCS, Pune, India using a forward primer (5′-CGAGATCTATGACCGCCCCGATCTGGAT-3′) and a reverse primer (5′-GCGAATTCTCACTCCACCCGGGTCGCTGA-3′) appended with BglII and EcoRI sites (underlined) as described earlier^28^. The amplicon was cloned into the BglII and EcoR1 site of a mammalian expression vector pEGFP-C1 (Clontech, Palo Alto, CA, USA) in frame with the EGFP protein. The recombinant clones were confirmed by sequencing and expression of the recombinant protein was confirmed by Western blot analysis using anti-GFP (Sigma-Aldrich, USA) and anti-PPE2 antibody raised in mouse and anti-mouse IgG-HRP (Sigma-Aldrich, USA) was used as secondary antibody. For affinity purification of the recombinant PPE2 protein, the gene was amplified from BAC-Rv329 (A5) using a forward primer (5′-CGAGAGCTCATGACCGCCCCGATCTGGATGG-3′) and a reverse primer (5′-GCAGAATTCTCACTCCACCCGGGTCGCTGAGT-3′) and cloned into the SacI and EcoR1 site of a T7 polymerase driven *Escherichia coli* expression vector pRSET A (Invitrogen, Carlsbad, CA, USA) in frame with an N-terminal histine tag to facilitate purification using metal affinity purification resins. All the recombinant clones were confirmed by PCR and sequencing and expression of the recombinant protein was confirmed by SDS-PAGE analyses after induction. The pRSET A vector containing the PPE2 gene was transformed into *E. coli* BL21 (DE3) pLysS cells and a primary inoculum was prepared from a single transformed colony. The primary culture was inoculated into 800 ml terrific broth in the presence of chloramphenicol (35 μg/ml) and ampicillin (100 μg/ml). The culture was grown in a shaker incubator till the absorbance reached to about 0.5. Expression of the recombinant protein was induced by adding IPTG to a final concentration of 1 mM and incubation was continued further for 16 hours at 18 °C in a shaker incubator to favor soluble expression of the protein. Cells were then harvested by centrifugation and the pellets were suspended in lysis buffer (PBS containing 5% glycerol, 0.3% sodium lauroyl sarcosine). The cell suspension was then sonicated and the lysate was centrifuged at 12000 rpm for 30 minutes. The soluble recombinant protein present in the supernatant was allowed to bind with the TALON resin (Clontech, USA) for an hour, loaded into a column and washed with washing buffer (PBS containing 5% glycerol and 20 mM Imidazole). The resin-bound protein was eluted using elution buffer (PBS containing 5% glycerol and 200 mM imidazole). Eluted samples were loaded onto an SDS-PAGE gel to confirm the presence of the recombinant PPE2 protein. The PPE2-positive fractions were pooled together and dialyzed against PBS containing 5% glycerol to remove imidazole from the protein samples. The concentration of the purified protein was estimated using Micro BCA^TM^ Protein Assay Kit from Thermo Scientific (Rockford, IL, USA) following manufacturer’s instructions.

### Generation of NLS mutants of PPE2 using site directed mutagenesis

Site directed mutagenesis was performed to generate NLS mutants of PPE2 using QuickChange^TM^ Site-Directed Mutagenesis Kit (Strategene, CA, USA) according to manufacturer’s protocol. A forward primer (5′-GCCCGAGGAACAGGTTCAACCGCAGCTCGGCCGCGGTTACGAATACCTGG-3′) and a reverse primer (5′-CTGCGGTTGAACCTGTTCCTCGGGCCCAGGTATTCGTAACCGCG GCCGAG-3′) were used to delete the nuclear localization signal (NLS) in order to obtain NLS-truncated mutant ΔNLS-PPE2. The amino acids in the predicted monopartite NLS (RRRRPKIKQ) were numbered from 1–9 respectively (R^1^R^2^R^3^R^4^P^5^K^6^I^7^K^8^Q^9^). Functionally important positively charged amino acids were mutated to neutral alanine residues. For stepwise mutagenesis following primer sets were used-

A. For mutations in position 4 and 6:

Mut 4, 6 Forward: 5′-CGCAGCGGCGTCGGGCACCAGCAATCAAACAGCTCGGC-3′

Mut 4, 6 Reverse: 5′-GCCGAGCTGTTTGATTGCTGGTGCCCGACGCCGCTGCG-3′

B. For mutations in position 1, 2 and 3 Mut 1, 2, 3 Forward: 5′ GAACAGGTTCAACCGCAGGCGGCTGCGGCACCAGCAATCAAAC-3’ Mut 1, 2, 3 Reverse: 5′-GTTTGATTGCTGGTGCCGCAGCCGCCTGCGGTTGAACCTGTTC-3′. These primers were used sequentially to generate the NLS mutant protein (Mut-NLS-PPE2) where all the 1, 2, 3, 4, 6 positions containing arginine (R) and lysine (K) residues were replaced by alanine (A) residues. All the mutant clones were confirmed by automated DNA sequencing.

### Generation of PPE2-specific polyclonal antibody in mice

Polyclonal antibody was generated against the full length PPE2 protein in BALB/c mice. The experimental protocols were approved by and performed as per the guidelines of the Institutional Animal Ethics Committee (IAEC) of Vimta Labs Ltd, Hyderabad (protocol number: PCD/CDFD/15). Mice were immunized with 100 μg of recombinant PPE2 protein in incomplete Freund’s adjuvant subcutaneously. Two booster doses of the recombinant protein (100 μg/animal) in incomplete Freund’s adjuvant were injected subcutaneously at 21 day intervals. Mice were sacrificed at day 75 and sera were collected. The sera were checked for the presence of PPE2 specific antibody by Western blotting. Whole cell extracts from clinical and laboratory strains of *M. tuberculosis* were used to determine the specificity of the generated antibodies.

### Cell culture

Peritoneal macrophages were harvested from C57BL/6 mice (about 4–6 week old) maintained in the animal house facility of Vimta Labs Ltd, Hyderabad. In brief, mice were injected intraperitoneally with 1 ml of 4% thioglycolate and sacrificed after 4 days using CO_2_ asphyxiation. The adherent macrophages were cultured with Dulbecco’s Modified Eagle Medium (DMEM) (Hyclone, GE Healthcare, Pittsburg, USA) containing 10% FBS and 1X antibiotic-antimycotic solution and 1X MEM vitamin (all from Invitrogen, Carlsbad, USA). The RAW 264.7 macrophages were cultured in DMEM (Hyclone) containing 10% FBS, 1X antibiotic-antimycotic solution and 1X MEM vitamin.

### Generation of stable transfectants of RAW 264.7 macrophages

The wild-type PPE2 as well as the NLS-mutants of PPE2 constructed earlier were cloned into pCX4neo vector at EcoRI and HpaI restriction sites with a 3X FLAG-tag appended to the forward primer. These pCX4neo clones containing PPE2 and its mutants were co-transfected in PLAT-E cells using Lipofectamine 2000 along with VSVG and VSVG-P vectors. The medium was changed after 5 hours and the culture was left for 70 hours. The supernatant containing recombinant viruses were collected and filtered through 0.44 μm filter. The RAW 264.7 macrophages pre-treated with 5 μg/ml polybrene for 1 hour were infected with these viruses and the positive clones were selected using geneticin (G418 sulphate, Invitrogen). The stable cells were frozen in 10% DMSO in FBS. The protein expression was confirmed using anti-FLAG monoclonal antibody (Sigma-Aldrich, USA) as well as with anti-PPE2 polyclonal antibody.

### Cloning and transformation of PPE2 and NLS-mutants of PPE2 in *Mycobacterium smegmatis*

The PPE2 open reading frame was amplified from BAC-Rv329 (A5), and cloned and expressed in *M. smegmatis* strain mc^2^155 as described previously^45^. The clones were confirmed by restriction digestion and sequencing. The expression of protein was confirmed by Western blotting using polyclonal antibody generated against PPE2.

### Measurement of Nitric oxide (NO) in culture supernatants

The accumulated nitrite resulting from NO production by the stimulated macrophages in culture was measured using Griess reagent. Briefly, 100 μl of Griess reagent [1% sulfanilamide in 2.5% H_3_PO_4_ and 0.1% naphtylethylenediamine in 2.5% H_3_PO_4_] was added to 100 μl of culture supernatants as described earlier^46^. The absorbance was measured at 570 nm in a spectrophotometer. The nitrite content in the samples was calculated based on a standard curve prepared from known concentrations of sodium nitrite.

### Semi-quantitative reverse transcriptase polymerase chain reaction (RT-PCR)

Total RNA was isolated from the RAW 264.7 macrophages using Trizol reagent (Invitrogen, Carlsbad, CA, USA) following the manufacturer’s instructions. Reverse transcription was carried out using 2 μg of total RNA using Moloney Murine Leukemia Virus (MMLV) reverse transcriptase (Invitrogen). Semi-quantitative RT-PCR was performed using *inos* specific primer pairs provided in a gene-specific relative RT-PCR kit (Ambion, Austin, USA). 18 S rRNA was used as an internal control using the primers supplied with the kit. For detecting *inos* transcripts, RT-PCR was performed using *inos* gene specific primers (forward, 5′-TCCAGAAGCAGAATGTGACC-3′ and reverse, 5′-GGACCAGCCAAATCCAGT-3′ to amplify 120 bp region) and a house keeping gene β-actin (forward, 5′-GTGGGCCGCTCTAGGCACCA-3′ and reverse, 5′-CGGTTGGCCTTAGGGTTCAGGGGGG-3′ to amplify 244 bp region) was used as an internal control.

### Infection of macrophages and CFU count

About 0.5 million macrophages were infected for 4 hours with *M. smegmatis* strains harbouring either the wild-type PPE2 or ΔNLS-PPE2 or the backbone vector (pVV16) at 10 multiplicities of infection. The free suspended bacteria were removed by washing the cells with DMEM. Cells were harvested at various time points and lysed in 0.1% Triton X-100 containing 1X PBS. Serial dilutions of cell extracts were plated on 7H10 plates supplemented with 10% ADC (HIMEDIA, India) and 25 μg/ml Kanamycin, 50 μg/ml Hygromycin. Plates were incubated at 37 °C and colonies were counted after 4–6 days as described earlier^47^.

### *In vivo* infection studies

Bacterial loads in liver, lung and spleen were evaluated at different time points after intraperitoneal infection of 4–6 weeks old mice C57/BL6, maintained in animal house facility of Vimta Labs, Hyderabad, with 50 × 10^6^ *M. smegmatis* bacteria expressing either the wild-type PPE2 or ΔNLS-PPE2 or harbouring the backbone vector (pVV16). After day 2 and day 7, lung, liver and spleen were aseptically removed from euthanized animals from each group. Organs were homogenized in sterile saline containing 0.05% Tween 80 (Sigma-Aldrich, USA) and plated on 7H10 plates supplemented with 10% ADC (HIMEDIA, India) and 25 μg/ml Kanamycin, 50 μg/ml Hygromycin to count bacterial colonies as described earlier^47^. Experiments were performed as per the guidelines and protocols approved by the Institutional Animal Ethics Committee.

### Confocal microscopy

RAW 264.7 macrophages were transfected with the wild-type (EGFP-PPE2) or mutant constructs (EGFP-MutNLS-PPE2, EGFP-ΔNLS-PPE2) in 4 chamber glass slides (BD Falcon, Bedford, MA USA) or on coverslips. In some experiments after 5 hours of transfection, cells were treated with 10 μM ivermectin. At 24 hours post-transfection, cells were fixed in 1% paraformaldehyde for 10 minutes and washed 5 times with PBS. The fixed cells were then mounted in Vectashield mounting medium containing DAPI (Vector Laboratories, Inc. Burlingame CA). The nuclear transport of GFP-tagged PPE2 was visualized under LSM 510META confocal microscope using 40X oil-immersion objective (Carl Zeiss, Jena, Germany). To detect GFP, the cells were excited at 488 nm and emission was recorded using 505 nm long pass filter.

### Electrophoretic mobility shift assay (EMSA)

EMSA was carried out as described earlier^48^. For EMSA analyses, the following oligonucleotides were used as probe or competitor: NF-κB consensus [AGTTGAGGGGACTTTCCCAGGC], IRF-1 consensus [CACTGTCAATATTTCACTTTCATAATG], GATA consensus [CACTTGATAACAGAAAGTGATAACTCT], *inos* specific putative GATA-1 binding element [(proximal) GGGGTATAAATACCTGATGGCTGCTGCCAG], *inos* specific proximal NF-κB [CCAACTGGGGACTCTCCCTTTGGGAACA]. The single stranded oligonucleotides were annealed with the respective complimentary oligonucleotides by incubating at 95 °C for 10 minutes in 100 mM NaCl and gradually cooling to room temperature. The double stranded oligonucleotides were end-labeled with [γ^32^P]-ATP using T4 polynucleotide kinase. For competition studies, 100-fold molar excess of the unlabeled oligonucleotides was incubated along with the labeled probes. EMSA was performed as described earlier. Briefly, different amounts of recombinant PPE2 or nuclear extracts prepared from RAW 264.7 macrophages stably expressing vector (pCX4neo) or wild-type PPE2 (pCX4neo-PPE2) following the protocol as described earlier^45^,^48^ were incubated with 1 ng of ^32^P–end-labelled oligonucleotide in a binding buffer (20 mM HEPES, 0.5 mM DTT, 0.5 mM EDTA, and 5% glycerol for 30 minutes at room temperature). The DNA-protein complex was resolved on a 7% native polyacrylamide gel using TGE running buffer (25 mM Tris, 190 mM Glycine, 1 mM EDTA, pH 8.3). The specificity of the binding was determined by competition with 100-fold molar excess of unlabeled probe. For supershift assay, Protein A-purified mouse anti-PPE2 antibody was added to the binding reaction to a final concentration of 10 μg per binding reaction. The gel was dried at 80 °C for 1 hour and exposed to imaging plate (FujiFilm) overnight. Visualization of the radioactive bands was carried out using STARION phosphor imager (FujiFilm FLA-9000).

### Luciferase and β-Galactosidase reporter assays

About one million RAW 264.7 macrophages stably expressing control vector, wild-type PPE2 or ΔNLS-PPE2 were transfected with 2.5 μg *inos*-luc (Kind gift from Prof. E.D Chan, University of Colorado, Denver) and 0.5 μg pCDNA-LacZ plasmids using Lipofectamine LTX along with plus reagent (3 μg) and at 8 hour post-transfection, cells were stimulated with 3 μg/ml LPS plus 10 ng/ml IFN-γ. Cells harvested at 24 hour post-stimulation were lysed with reporter lysis buffer (Promega, Madison, WI, USA) and protein concentration was estimated with Micro BCA^TM^ Protein Assay Kit following the manufacturer’s instructions. About 50 μg protein in 20 μl lysis buffer was mixed with 100 μl of luciferase substrate reagent (Promega). The light emitted was measured using TD 20/20 luminometer (Turner Biosystems, CA, USA). For β-Galactosidase reporter assay, 100 μg of protein was used. The β-Galalactosidase was quantitated using β-Galactosidase reporter assay kit (Roche Applied Science) following the manufacturer’s protocol as described earlier^45^. The results were expressed as relative luciferase units normalized to β-Galalactosidase activity.

### Pull down assay

One mg of RAW 264.7 macrophages lysate prepared using whole cell lysis buffer (Tris-HCl pH 7.4 20 mM, NaCl 150 mM, Triton X-100 1%, Sodium pyrophosphate 2.5 mM, β-Glycerophosphate 1 mM, EDTA 1 mM, EGTA 1 mM, Na_3_VO_4_ 1 mM, PMSF 1 mM, protease inhibitor cocktail) was incubated overnight with 20 μl bed volume of the Talon beads bound to recombinant full-length or mutant PPE2 protein. The beads were spinned down and washed 5 times with whole-cell lysis buffer. The washed beads were resuspended in 100 μl of the lysis buffer and boiled with 6X loading dye and Western blotting was performed using anti-importin-α antibody (Sigma-Aldrich, USA). The blot was reprobed with anti-PPE2 antibody. About 50 μg of the lysate from each group was used as input control.

### Reverse transcription qPCR for *inos* mRNA quantification

Reverse transcription qPCR was carried out as described earlier^49^,^50^. In brief, total RNA was isolated from various tissues of the infected animals using TRI Reagent (Sigma-Aldrich, Bangalore, India) as per manufacturer’s instructions. RNA concentration and purity was assessed using a Nanodrop ND-1000 spectrophotometer (Nanodrop Technologies, DE, USA). Quality of the isolated RNA was assessed by measuring the RIN (RNA integrity number) value using the RNA 6000 Nano LabChip Series II Assay in a 2100 Bioanalyzer System (Agilent Technologies, Palo Alto, CA, USA). About 2 μg total RNA with RIN value of >7.5 were used for generation of first strand cDNA using First Strand cDNA synthesis kit (Thermo Scientific, Waltham, MA USA) using OligodT_18_ primers as per manufacturer’s instructions. The cDNA reactions were diluted to 50 μl in nuclease-free water (Invitrogen Life Technologies), aliquoted and stored at −20 °C till further use.

Real time qPCR reactions were carried out in duplicate for each sample using SYBR Premix Ex Taq (Tli RNaseH Plus, DSS Takara Bio India Pvt. Ltd, India) in a 20 μl reaction volume containing each primers at 500 nM final concentration. Reactions were temperature cycled, and SYBR green levels were measured using a CFX-96 system (Bio-Rad Laboratories, Berkeley, California). The initial target concentration for each gene was calculated by relative standard curve method using a pool of experimental sample cDNA as calibrator. The level of expression for *inos* mRNA in each sample was normalized to corresponding GAPDH levels. All the primers were synthesized from Eurofins Genomics India Pvt. Ltd (Bangalore, India). The sequences of the primers for mouse *inos* and g*apdh* were used were as follows:

inos Forward Primer: 5′-GCCAATGAGGTACTCAGCGT-3′;

inos Reverse Primer: 5′-CTGCTCCTCGCTCAAGTTCA-3′

gapdh Forward Primer: 5′-ACAACTTTGTCAAGCTCATTTCC-3′

gapdh Reverse Primer: 5′-GATAGGGCCTCTCTTGCTCA-3′

The annealing temperatures for *inos* and *gapdh* primer pairs were 60 °C and 56 °C respectively.

### Statistical analyses

For multiple group comparisons, one way ANOVA test was performed. Individual statistics of unpaired samples was performed by the t-test. Calculations were performed using GraphPad Prism, version 5.02. *p* < 0.05 was considered to be significant.

## Additional Information

**How to cite this article**: Bhat, K. H. *et al*. The PPE2 protein of *Mycobacterium tuberculosis* translocates to host nucleus and inhibits nitric oxide production. *Sci. Rep.* **7**, 39706; doi: 10.1038/srep39706 (2017).

**Publisher's note:** Springer Nature remains neutral with regard to jurisdictional claims in published maps and institutional affiliations.

## Supplementary Material

Supplementary Figures
