# Supplementary material for: The PPE2 protein of Mycobacterium tuberculosis translocates to host nucleus and inhibits nitric oxide production
Source: Sci Rep. 2017 Jan 10;7:39706. doi: 10.1038/srep39706 (PMC5223167; doi:10.1038/srep39706)
Supplement: Supplementary Figures [file srep39706-s1.pdf]

The PPE2 protein of *Mycobacterium tuberculosis* translocates to host nucleus and inhibits nitric oxide production

Khalid Hussain Bhat[1,2,3], Shruti Srivastava[1,2,3], Sandeep Kumar Kotturu[4], Sudip Ghosh[4] and Sangita Mukhopadhyay[1,*]

[1]Laboratory of Molecular Cell Biology, Centre for DNA Fingerprinting and Diagnostics (CDFD), Tuljaguda Complex, Nampally, Hyderabad, India
[2]Graduate Studies, Manipal University, Manipal, Karnataka, India
[3]Equal authorship
[4]Molecular Biology Division, National Institute of Nutrition (ICMR), Jamai-Osmania PO, Hyderabad, India

***Address for correspondence and reprint requests to** Sangita Mukhopadhyay, Laboratory of Molecular Cell Biology, Centre for DNA Fingerprinting and Diagnostics (CDFD), Tuljaguda Complex, Nampally, Hyderabad, India. E-mail address: sangita@cdfd.org.in, sangitacdfd@gmail.com

Running title: PPE2 inhibits NO production

A

S. no.	ORF	Gene	NLS motif	DNA binding signal
1.	Rv0096	ppe1	-	-
2.	Rv0256c	ppe2	+	+
3.	Rv0280	ppe3	-	-
4.	Rv0286	ppe4	-	-
5.	Rv0304c	ppe5	-	-
6.	Rv0305c	ppe6	-	-
7.	Rv0354c	ppe7	-	-
8.	Rv0355c	ppe8	-	-
9.	Rv0388c	ppe9	-	-
10.	Rv0442c	ppe10	-	-
11.	Rv0453	ppe11	-	-
12.	Rv0755c	ppe12	-	-
13.	Rv0878c	ppe13	-	-
14.	Rv0915c	ppe14	-	-
15.	Rv1039c	ppe15	-	-
16.	Rv1135c	ppe16	-	-
17.	Rv1168c	ppe17	-	-
18.	Rv1196	ppe18	-	-
19.	Rv1361c	ppe19	-	-
20.	Rv1387	ppe20	-	-
21.	Rv1548c	ppe21	-	-
22.	Rv1705c	ppe22	-	-
23.	Rv1706c	ppe23	-	-
24.	Rv1753c	ppe24	-	-
25.	Rv1787	ppe25	-	-
26.	Rv1789	ppe26	-	-
27.	Rv1790	ppe27	-	-
28.	Rv1800	ppe28	-	-
29.	Rv1801	ppe29	-	-
30.	Rv1802	ppe30	-	-
31.	Rv1807	ppe31	-	-
32.	Rv1808	ppe32	-	-
33.	Rv1809	ppe33	-	-
34.	Rv1917c	ppe34	-	-
35.	Rv1918c	ppe35	-	-
36.	Rv2108	ppe36	-	-
37.	Rv2123	ppe37	+	-
38.	Rv2352c	ppe38	-	-
39.	Rv2353c	ppe39	-	-
40.	Rv2356c	ppe49	-	-
41.	Rv2430c	ppe41	-	-
42.	Rv2608	ppe42	-	-
43.	Rv2768c	ppe43	-	-
44.	Rv2770c	ppe44	-	-
45.	Rv2892c	ppe45	-	-
46.	Rv3018c	ppe46	-	-
47.	Rv3021c	ppe47	-	-
48.	Rv3022c	ppe48	-	-
49.	Rv3125c	ppe49	-	-
50.	Rv3135	ppe50	-	-
51.	Rv3136	ppe51	-	-
52.	Rv3144c	ppe52	-	-
53.	Rv3159c	ppe53	-	-
54.	Rv3343c	ppe54	-	-
55.	Rv3347c	ppe55	-	-
56.	Rv3350c	ppe56	-	-
57.	Rv3425	ppe57	-	-
58.	Rv3426	ppe58	-	-
59.	Rv3429	ppe59	-	-
60.	Rv3478	ppe60	-	-
61.	Rv3532	ppe61	-	-
62.	Rv3533c	ppe62	-	-
63.	Rv3539	ppe63	-	-
64.	Rv3558	ppe64	-	-
65.	Rv3621c	ppe65	-	-
66.	Rv3738c	ppe66	-	-
67.	Rv3739c	ppe67	-	-
68.	Rv3873	ppe68	-	-
69.	Rv3892c	ppe69	-	-

B

MTAPIWMASPPEVHSALLSSGPGPGPLLVSAEGWHSLSIAYAETADELAALL
AAVQAGTWDGPTAAVYVAAHTPYLAWLVQASANSAAMATRQETAATAYGT
ALAAMPTLAELGANHALHGVLMATNFFGINTIPIALNESDYARMWIQAATTMA
SYQAVSTAAVAAAPQTTPAPQIVKANAPTAASDEPNQVQEWLQWLQKIGYT
DFYNNVIQPFINWLTNLPFLQAMFSGFDPWLPSLGNPLTFLSPANIAFALGYP
MDIGSYVAFLSQTFAFIGADLAAAFASGNPATIAFTLMFTTVEAIGTIITDTIALV
KTL**LEQTLALLPAALPLLAAPLAPL**TLAPASAAGGFAGLSGLAGLVGIPPSAP
PVIPPVAAIAPSIPTPTPTPAPAPAPTAVTAPTPPPGPPPPPVTAPPPVTGAGI
QSFGYLVGDLNSAAQARKAVGTGVRKKTPEPDSAEAPAAAAAPEEQVQPQ
RRRRPKIKQLGRGYEYLDLDPETGHDPTGSPQGAGTLGFAGTTHKASPGQ
VAGLITLPNDAFGGSPRTPMMPGTWDTDSATRVE

Figure S1: Primary amino acid sequence analyses of PPE proteins of *M. tuberculosis*. (A) Analysis of all the PPE proteins of *M. tuberculosis* reveals that among the PPE members only PPE2 contains both DNA binding domain and a nuclear localization signal (NLS). An NLS motif was predicted in PPE37 but no DNA-binding domain was predicted. (**B**) Location of the predicted monopartite nuclear localization signal (underlined in green) and leucine zipper motif (underlined in red) in the primary amino acid sequence of PPE2 protein of *M. tuberculosis*.

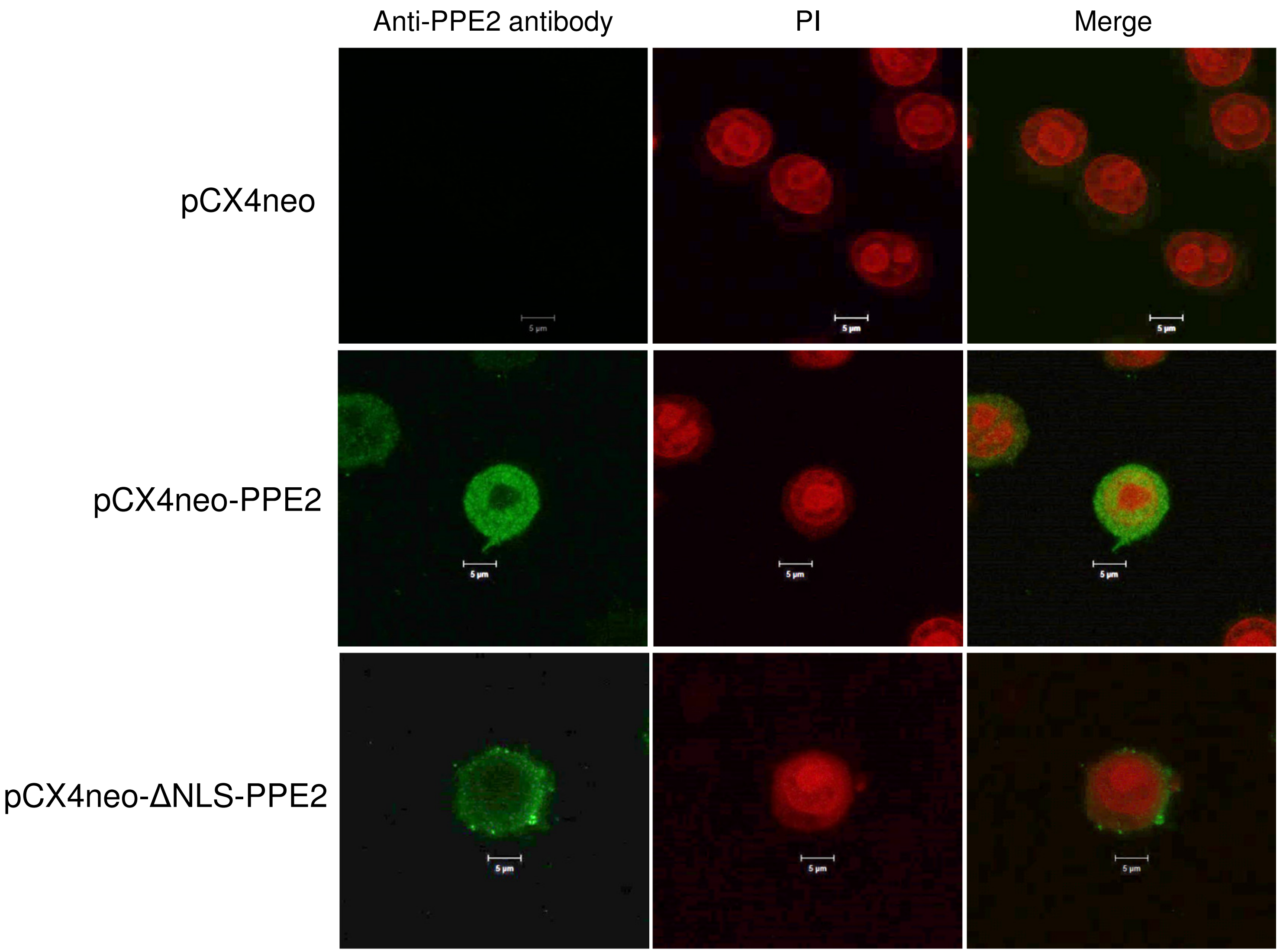

Figure S2. Translocation of PPE2 in the nucleus of RAW 264.7 macrophages stably expressing wild-type PPE2. RAW 264.7 macrophages stably expressing backbone vector (pCX4neo) or wild-type PPE2 (pCX4neo-PPE2) or ΔNLS-PPE2 (pCX4neo-ΔNLS-PPE2) were fixed using 1% paraformaldehyde and then permeabilized with 0.2 % Triton X-100 in PBS. Cells were washed and blocked. Nuclear localization of PPE2 (green) was detected using mouse anti-PPE2 antibody plus anti-mouse antibody conjugated to FITC by confocal microscopy. Nucleus was visualized by staining with Propidium iodide (PI). Scale bar 5 μM.

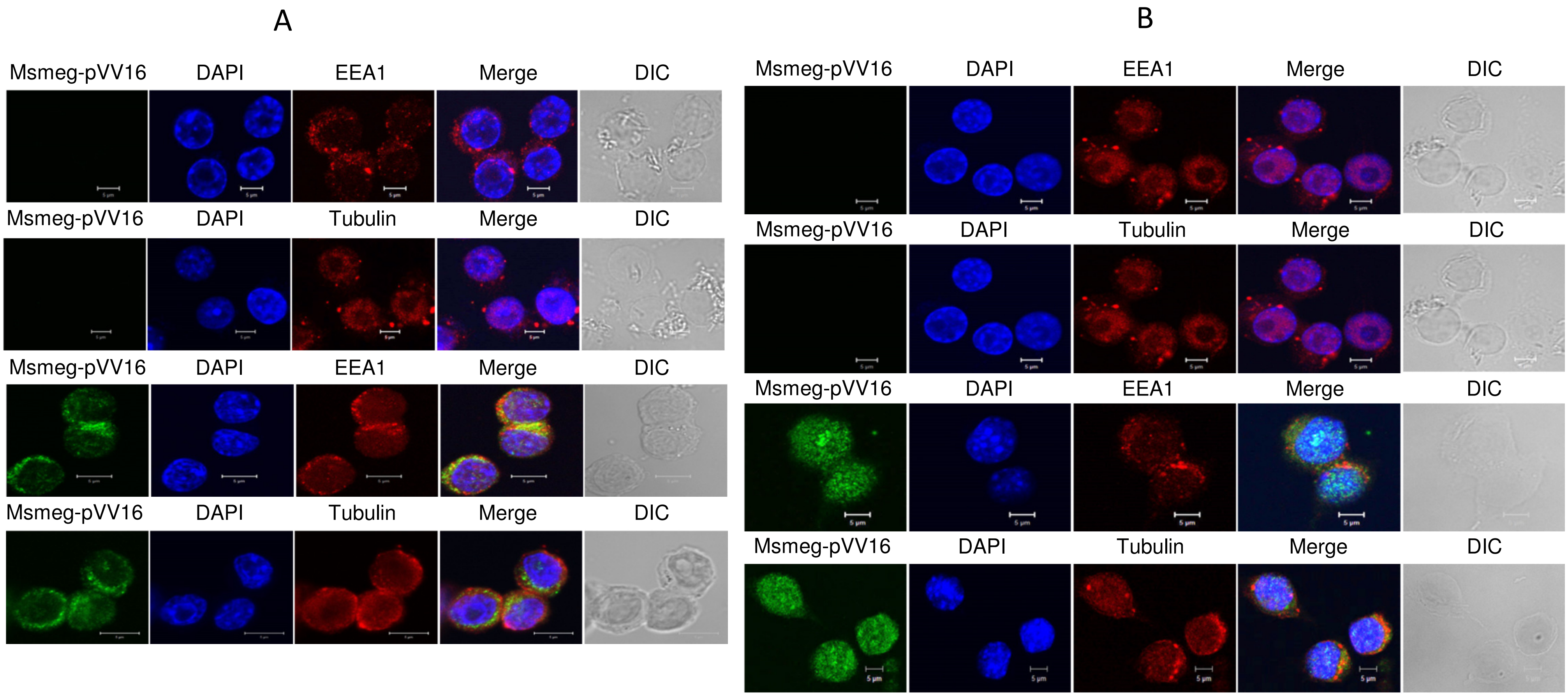

Figure S3. PPE2 is localized predominantly in endosome at 1 hour but in cytosol and nucleus at 6 hours after infection of RAW 264.7 macrophages. RAW 264.7 macrophages were infected with *M. smegmatis* strains carrying either backbone vector (Msmeg-pVV16) or wild type PPE2 (Msmeg-PPE2) at 1:100 MOI and after 1 hour (**A**) and 6 hours (**B**) post-infection, cells were fixed and processed for immunofluorescence using either mouse anti-PPE2 antibody or rabbit anti-EEA1 antibody or rabbit anti-alpha-tubulin antibody followed by incubation with either anti-mouse Alexa 488 (green) or anti-rabbit Alexa 596 (red). Samples were viewed with a Zeiss LSM 510 confocal microscopy. Nucleus was visualized by staining with DAPI. Data are representative of 3 independent experiments.

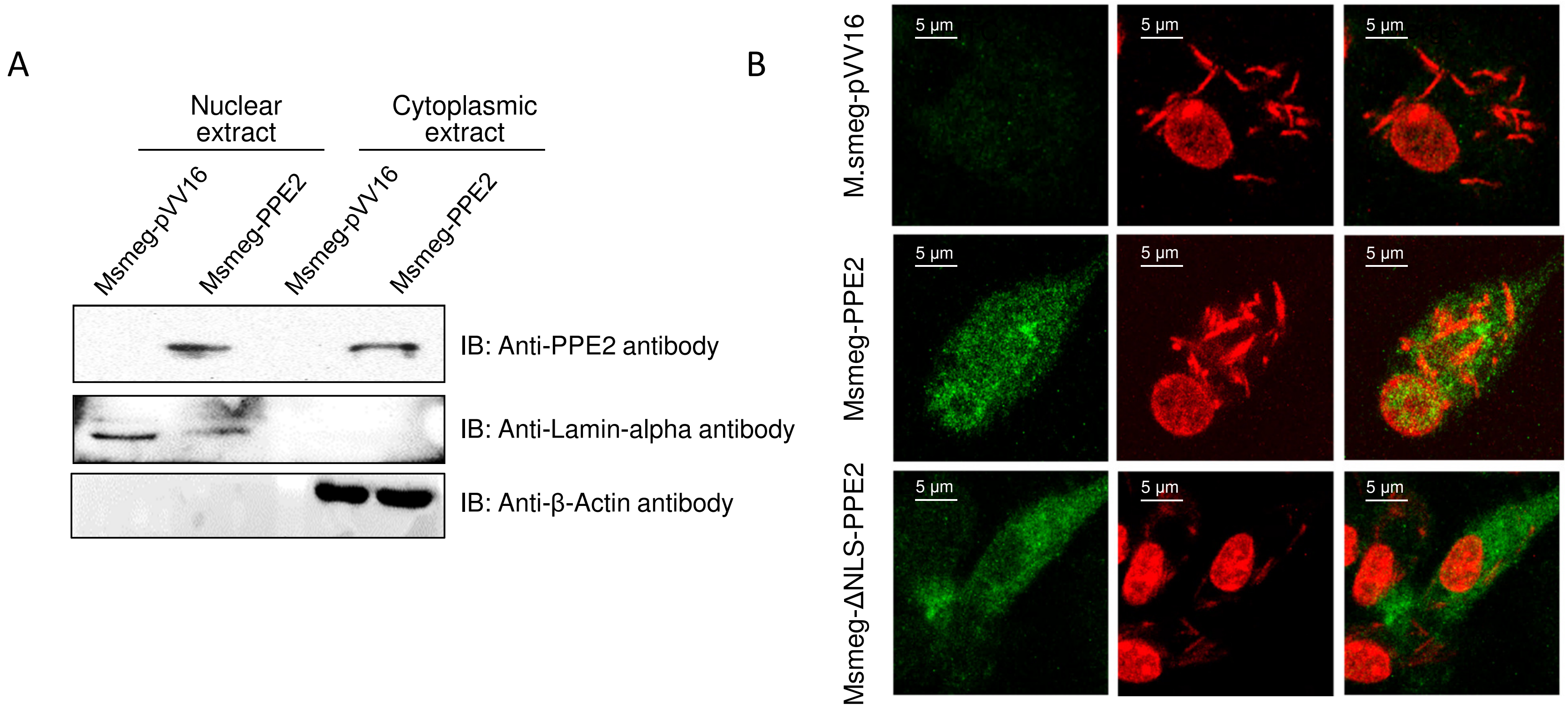

Figure S4: PPE2 protein is localized in the nucleus during infection of macrophages with *M. smegmatis* expressing PPE2. (**A**) Thioglycolate elicited peritoneal macrophages from C57BL/6 mice were infected with either Msmeg-PPE2 or Msmeg-pVV16 and presence of PPE2 protein was checked in the nuclear extracts and cytoplasmic extracts using anti-PPE2 antibody plus anti-mouse antibody conjugated to HRP. Lamin-alpha and β-Actin was used as input control for nuclear extracts and cytoplasmic extracts respectively. Data are representative of 3 independent experiments. (**B**) In another experiment, peritoneal macrophages from C57BL/6 mice were infected with *M. smegmatis* strain carrying either the backbone vector (Msmeg-pVV16) or wild-type PPE2 (Msmeg-PPE2) or PPE2 with a truncated NLS (Msmeg-ΔNLS-PPE2) at 1:100 MOI. Cells were harvested after 24 hours post-infection and nuclear localization of PPE2 (green) was detected using mouse anti-PPE2 antibody plus anti-mouse antibody conjugated to FITC by confocal microscopy. Nuclei were stained with Propidium iodide (PI).

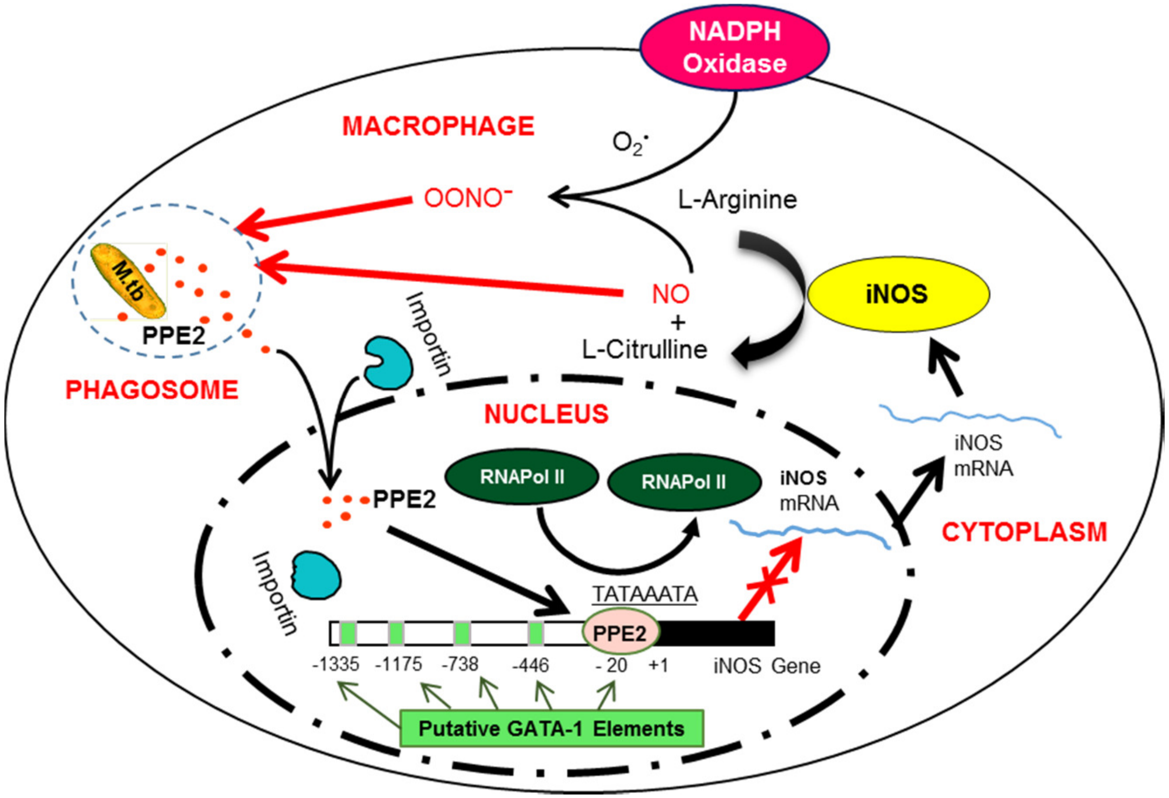

Figure S5: Schematic representation of the possible mechanism of inhibition of *inos* gene transcription by PPE2. Upon infection, PPE2 secreted by *M. tuberculosis* is leaked out to the cytoplasm from the phagosome, and is translocated to the host nucleus by utilizing the classical nuclear import machinery importin α/β. In the nucleus, PPE2 binds to the proximal GATA-1-binding element of the *inos* promoter that overlaps with the TATA box and directly competes with recruitment of RNA pol II transcription machinery to the promoter. This results in decreased levels of *inos* transcript leading to less amount of NO production.
